# Ocular Surface Development and Gene Expression

**DOI:** 10.1155/2013/103947

**Published:** 2013-02-21

**Authors:** Shivalingappa K. Swamynathan

**Affiliations:** Departments of Ophthalmology, and Cell Biology and Physiology, McGowan Institute of Regenerative Medicine, University of Pittsburgh School of Medicine, 203 Lothrop Street, Room 1025, Pittsburgh, PA 15213, USA

## Abstract

The ocular surface—a continuous epithelial surface with regional specializations including the surface and glandular epithelia of the cornea, conjunctiva, and lacrimal and meibomian glands connected by the overlying tear film—plays a central role in vision. Molecular and cellular events involved in embryonic development, postnatal maturation, and maintenance of the ocular surface are precisely regulated at the level of gene expression by a well-coordinated network of transcription factors. A thorough appreciation of the biological characteristics of the ocular surface in terms of its gene expression profiles and their regulation provides us with a valuable insight into the pathophysiology of various blinding disorders that disrupt the normal development, maturation, and/or maintenance of the ocular surface. This paper summarizes the current status of our knowledge related to the ocular surface development and gene expression and the contribution of different transcription factors to this process.

## 1. Introduction

**Figure 1:**
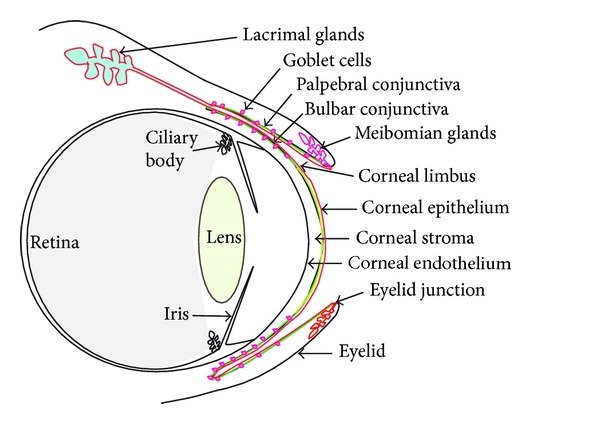
The ocular surface (shown as a thin red line) consists of the contiguous corneal (yellow) and conjunctival (green) surfaces bathed in the tear film and the associated ocular adnexa including the lacrimal and meibomian glands.

The ocular surface consists of a continuous epithelial surface with regional specializations, including the surface and glandular epithelia of the cornea, conjunctiva, lacrimal gland, accessory lacrimal glands, and meibomian gland, which are connected by the overlying tear film (Figure 1). The ocular surface serves a pivotal role in vision by ensuring scatter-free transmission of light and appropriate refraction. The transparent and refractive functions of the avascular cornea are supported by the tear film produced by the surrounding tissues of the ocular surface. The protective tear film is a complex fluid consisting of an inner mesh of transmembrane mucins on the surface epithelial cells soaked in a central aqueous layer secreted by the lacrimal glands and the outermost lipid layer secreted by the meibomian glands. The aqueous layer contains soluble mucins secreted by the conjunctival goblet cells, and soluble proteins and solutes secreted by the surface epithelial cells and plays a major role in protective functions of the tear film. Defective development and/or maintenance of the ocular surface is a leading cause of vision-related problems. It is estimated that as many as 285 million people worldwide are visually impaired, of whom 39 million are blind due to a variety of reasons [1]. Among them, corneal opacity accounts for about 4% of blindness, with trachoma causing another 3% [1].

**Figure 2:**
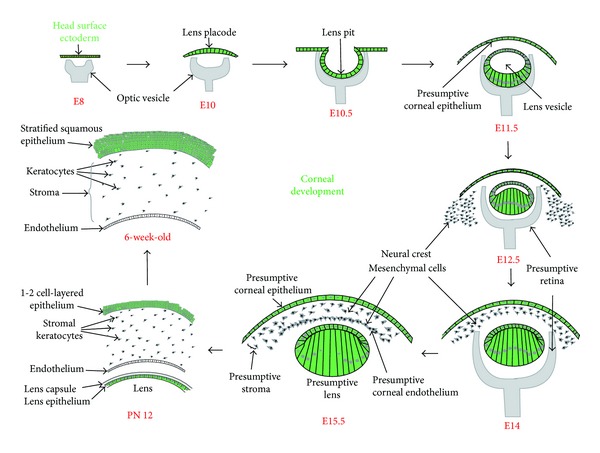
Mouse corneal development. Major events in mouse corneal development between embryonic day 8 (E8) and postnatal day 56 (PN56) are shown. Corneal epithelium and lens are derived from the head surface ectoderm (shown in green), while the corneal stroma and endothelium originate from the neural crest-derived mesenchymal cells.

Ocular surface development depends on a series of well-coordinated interactions between the neuroectoderm that forms the retina and the surface ectoderm that forms the lens and cornea, with important contributions from the neural crest-derived periocular mesenchymal cells (Figure 2) [2, 3]. One of the currently emerging themes in developmental ophthalmology is that the well-coordinated changes in gene expression accompanying ocular surface development are regulated by a combinatorial effect of a handful of transcription factors. This paper summarizes the current knowledge in regulation of gene expression during mouse ocular surface development. 

## 2. Development, Maturation, and Maintenance of the Ocular Surface

The cornea comprises of the anterior epithelium (stratified squamous cells derived from the surface ectoderm), central stroma (a thick collagenous mass of extracellular matrix with scattered neural crest-derived keratocytes), and the posterior endothelium (a monolayer of neural crest-derived cells) (Figure 2) [4, 5]. In the mouse, early eye development begins with the formation of the lens placode around embryonic day 10 (E10) (Figure 2). The lens vesicle is formed around embryonic day 11 (E11) by invagination of the lens placode in response to signals from the underlying optic vesicle (Figure 2). The overlying head surface ectoderm forms the presumptive corneal epithelium, which remains 1-2 cell layered at birth. Following eyelid opening around postnatal (PN) day 12, 1-2 cell-layered corneal epithelial cells divide rapidly and differentiate to form the 5-6 cell-layered epithelium by PN21 and a mature 6–8 cell-layered stratified squamous epithelium by about 8–10 weeks after birth. Around E12.5, neural crest-derived mesenchymal cells that surround the developing eye begin to migrate in between the surface ectoderm and the lens vesicle, forming the presumptive stroma and the corneal endothelium by E15.5 (Figure 2). Note that the term corneal “endothelium” is a misnomer, as it consists of neural crest-derived cells rather than endodermis-derived cells. The corneal stromal cells produce and secrete an extracellular matrix comprising of collagen fibrils and a variety of proteoglycans, the precise organization of which ensures corneal curvature, transparence, and mechanical strength. In postnatal stages, the corneal stromal cell density decreases gradually. The monolayer of endothelial cells that line the posterior of the cornea form tight junctions and help maintain stromal hydration by pumping excess water from the stroma into the anterior chamber. 

Though most features of the mature cornea are evident by about 6 weeks of age [4, 5], it is still debated as to when the fully mature cornea is formed. Proliferation and differentiation of the corneal epithelial cells continue in the adult mouse, allowing the sloughed-off superficial epithelial cells to be steadily replaced by differentiation of the slow-dividing basal cells, which in turn are replenished by stem cells originating from the corneal limbal epithelium [6–8]. The corneal epithelial cells accumulate high levels of taxon-specific corneal crystallins aldehyde dehydrogenase 3A1 (Aldh3a1) and transketolase (Tkt), which account for about 50% and 10% of water-soluble proteins, respectively [9–11], along with structural proteins such as keratin-12 [12]. Though it is suggested that the corneal crystallins are essential for the transparent and refractive properties of the cornea, convincing evidence supporting such roles is still scanty [10].

A fully functional lacrimal system is in place by the time of mouse eyelid opening around PN12, when the cornea is first exposed to the environment. The mouse lacrimal gland formation begins with a bud-like invagination of the temporal conjunctival forniceal epithelium around E13, which starts branching around E15.5 [13]. The meibomian gland buds, on the other hand, are apparent around E18.5 with ductal branching first detected at PN5 [14]. Both FGF10 signaling and Pax6 play important roles in lacrimal gland formation [13, 15]. Branching and differentiation of both lacrimal and meibomian glands is complete by around eyelid opening. Conjunctival goblet cells, which produce and secrete soluble mucins to the tear film, also first appear around PN12 [16], meeting the final physiological requirement for a fully functional lacrimal system before the eyelids open.

## 3. Differentially Expressed Genes in Different Components of the Ocular Surface

Several attempts have been made to identify differentially expressed genes in the mature mouse cornea, conjunctiva, and the limbus located in between. The basal layer of limbal epithelium located between the conjunctiva and the cornea is enriched in stem cells that serve as a source for transient-amplifying cells which migrate to the central corneal epithelium. Comparison of the rat limbal and central corneal transcripts by serial analysis of gene expression (SAGE) identified 759 transcripts specific for the limbus and 844 transcripts specific for the central cornea, with 2292 transcripts present in both [17]. A comparative analysis of the human corneal and conjunctival epithelial gene expression identified 93 and 211 transcripts exclusive to corneal and conjunctival epithelium, respectively. Biological processes related to cell adhesion, redox equilibria, and cytoprotection were overrepresented in the cornea, while innate immunity and melanogenesis were most prominent in the conjunctiva [18]. Microarray analysis of the pig limbal side population cells (enriched in stem cells) identified the genes responsible for the slow cycling and low metabolic activity of the limbal stem cell population [19, 20]. In another study, laser capture microdissection followed by microarray analysis identified about 100 differentially expressed genes in the mouse limbal compared to corneal epithelial basal cells [21]. Together, these studies identify differential gene expression profiles in these adjacent tissues and provide valuable insights related to the ocular surface cell biology.

Gene expression in the lacrimal glands has been the target of a few recent investigations. Large-scale sequencing of cDNA libraries generated from mouse and human lacrimal glands revealed significant differences suggestive of molecular divergence between the two species [22]. Laser capture microdissection coupled with microarray analysis demonstrated polarized expression of transporters and channels in lacrimal gland duct cells, consistent with the relatively high K+ in lacrimal fluid [23]. Differential gene expressions in the lacrimal gland during development of dry eye in a mouse model of Sjögren's syndrome-like disease identified 552 differentially expressed genes, providing new insight into the underlying cause or regulation of Sjögren's syndrome [24]. Further analysis in these mice-revealed alterations in tight junctions, adherens junctions, desmosomes, and gap junctions, suggesting perturbations in the permeability of the paracellular spaces between epithelial barriers in Sjögren's syndrome [25]. Gene expression arrays using total RNA isolated from the human accessory lacrimal glands (also called Glands of Wolfring) which secrete directly onto the ocular surface collected from frozen sections of eyelids by laser microdissection identified 24 most highly expressed genes, many of which were of direct relevance to lacrimal function [26]. The human accessory lacrimal glands are enriched in genes with antimicrobial activity and those related to protein synthesis and secretion, including ion channels and transporters, carbonic anhydrase, and aquaporins [26]. Genes encoding lysozyme, lactoferrin, tear lipocalin, and lacritin were among the most highly expressed in the human accessory lacrimal glands [26].

Similar large-scale profiling of transcriptomes has been attempted in meibomian glands as well. A study designed to identify the changes in gene expression associated with human meibomian gland dysfunction (MGD) identified about 400 genes with significant alterations [27]. In addition, this study revealed that the human meibomian gland gene expression signature is significantly different from that of the adjacent tissues [27]. Comparison of gene expression in lacrimal and meibomian glands obtained from ovariectomized mice treated with testosterone, estrogen, or control vehicle for 14 days revealed the sex-specific effects of sex steroids in the lacrimal and meibomian glands [28, 29]. Taken together, these studies identify differential gene expression profiles in the ocular adnexa and reveal the molecular basis for complex pathophysiological responses in tear film composition to sex hormones and during MGD.

## 4. Gene Expression during Corneal Development

Most large-scale studies of gene expression in corneas have attempted to characterize the early postnatal or adult mouse or rat corneal transcriptomes. No large-scale study has addressed the changes in gene expression patterns during human or mouse corneal embryonic development or in human corneas with developmental defects or diseases, presumably due to scarcity of tissues. Considering the important changes in mouse corneal morphology during post-eyelid-opening stages described above, a few studies have characterized the accompanying changes in corneal gene expression. Microarray analysis of using Affymetrix MG74Av2 chips targeting 8,666 unique characterized genes identified 442 genes differentially expressed between immature (PN10) and adult (PN49 to PN56) groups [30]. In a more thorough analysis, comparison of PN9 and 6-week-old adult mouse corneas by serial analysis of gene expression (SAGE) revealed dynamic changes in gene expression accompanying corneal postnatal maturation [31]. Roughly one third of the transcripts expressed in the PN9 or mature corneas were determined to be exclusive to each stage, with the remaining one third expressed at both stages [31]. 

By comparing the WT corneal transcriptomes at PN11 and PN56, we identified 1574 and 1915 genes whose expression decreased and increased, respectively, by more than 2-fold between PN11 and PN56 [32]. Apart from validating the previously identified changes in gene expression during corneal maturation [30, 31], this study identified additional genes whose expression was modulated in the cornea following eyelid opening. Transcripts encoding major extracellular matrix-(ECM-) related proteins were decreased between PN11 and PN56, suggesting that most of the stromal ECM is produced before or around eyelid opening. Consistent with the limited remodeling that occurs in the adult corneal stroma [33], Adam family proteinases and other MMPs required for remodeling ECM [34] were sharply decreased between PN11 and PN56. Expression of several cell-junctional complex components increased between PN11 and PN56, when the corneal epithelium stratifies. Similarly, several oxidative stress-related genes and solute carrier family members were upregulated between PN11 and PN56, reflecting the elevated oxidative stress and the need for solute transport in metabolically active adult corneas [32]. 

## 5. Gene Expression at the Corneal Limbus

The human corneal limbal epithelial crypt, an approximately 120 *μ*m long cord of cells, arises from the undersurface of interpalisade rete ridges of the palisades of Vogt in the corneoscleral limbus. Each human eye contains from 6 to 7 such limbal epithelial crypts randomly distributed along the limbus. It is largely accepted that the normal corneal epithelial homeostasis including epithelial wound healing and repopulating the cells sloughed off at the surface depends on normal division, migration, and stratification of the corneal epithelial stem cells that reside in the limbal epithelial crypts [35, 36]. A recent study challenged the notion that stem cells are limited to the corneal limbal area by demonstrating the presence of oligopotent stem cells dispersed throughout the cornea [37]. It is possible that these central corneal epithelial stem cells facilitate routine maintenance of the corneal epithelium while the limbal stem cells step in to repair acutely damaged corneal epithelium. In spite of this report, the clinical importance of corneal limbal stem cells is well established as judged by the successful use of limbal epithelial stem cell-derived cell sheaths in treating corneal defects [38–43]. Considering that the limbal epithelial stem cells serve as a useful resource for reconstruction of defective corneas, thorough understanding of the stem cell-enriched limbal epithelial gene expression patterns is necessary to create new opportunities for diagnostic and therapeutic interventions in severely damaged corneas [35].

In spite of their importance, identification of corneal limbal stem cell-specific markers has been a daunting challenge. Comparison of the rat limbal and central corneal epithelial transcriptomes by SAGE identified 759 limbal specific transcripts, 844 central corneal specific transcripts, and 2292 transcripts that were present in both regions [17]. Microarray comparison of transcriptomes from laser-microdissected limbal and central corneal basal epithelial cells revealed the identities of about 100 genes that were specifically expressed in the corneal limbus [21]. Microarray comparison of transcripts in the human limbal epithelial crypts with those in the cornea identified genes involved in cell cycling and self-renewal such as growth factors, cytokines, WNT, Notch, TGF-Beta pathways FZD7, BTG1, CCNG, and STAT3 as enriched in the cornea [44]. In contrast, genes involved in stem cell maintenance, such as cell adhesion molecules, WNT, and Notch-signaling pathway components, CDH1, SERPINF1, LEF1, FRZB1, KRT19, SOD2, and EGR1, were overexpressed in the limbal crypt [44]. Mitotically quiescent limbal stem cells may be identified by their coexpression of CCAAT enhancer binding protein-delta (C/EBP*δ*), Bmi1, and *δ*Np63*α* [45]. In addition, genes, including Krt15, Krt14, N-cadherin, cadherin-3, nestin, SOD2, Wnt4, Notch-1, SPON1, IFITM1, ITM2A, CXCR4, DKK4, NGF, and its receptor TrkA, have been proposed as corneal limbal basal epithelial stem cell-specific markers [46–52]. Wnt/*β*-catenin signaling is thought to regulate human corneal epithelial stem/progenitor cell proliferation [53]. Though a direct role has been proposed for transcription factors Pax6, *δ*Np63*α*, EGR1, TCF4, and C/EBP*δ* in maintenance of corneal limbal stem cell identity, involvement of other transcription factors controlling the limbal epithelial cell fate remains to be identified [45, 54, 55]. Though generally useful, these studies have failed to identify specific markers which could be used to define the corneal limbal stem cells at the molecular level. We are yet to arrive at a consensus regarding definitive markers for corneal limbal epithelial stem cells; the presence of which was first proposed more than two decades ago [56, 57]. 

## 6. Gene Expression in the Conjunctiva

**Figure 3:**
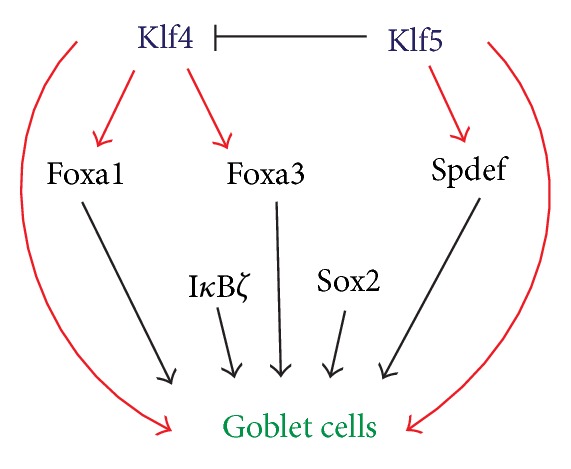
Hierarchical network of transcription factors regulating goblet cell development. Foxa1, Foxa3, and SPDEF, required for goblet cell development in other mucosal epithelia, are downregulated in *Klf4*CN and *Klf5*CN conjunctiva [16], placing Klf4 and Klf5 upstream of these factors in the network of factors governing goblet cell development.

Unlike the cornea, relatively few studies have addressed the changes in gene expression in the conjunctiva (Figure 3). In addition to the epithelial cells, conjunctiva contains goblet cells which produce and secrete mucins to the tear film. Conjunctival goblet cells play an important role in maintaining ocular surface homeostasis by producing and secreting mucins to the tear film [58]. In spite of their importance, relatively little is known about the factors regulating conjunctival goblet cells. Studies in other tissues have demonstrated the requirement of factors such as Foxa1, Foxa2, Foxa3, and Spdef for colonic and airway epithelial goblet cell development [59–63]. Studies in our laboratory have demonstrated that Klf4 and Klf5 are both required for conjunctival goblet cell development (Figure 3) [64, 65].

In order to identify the changes in postnatal mouse conjunctival forniceal gene expression and their regulation by Klf4 during the eye-opening stage when the goblet cells first appear, we used Laser microdissection (LMD) to collect conjunctival forniceal cells from PN 9, PN14, and PN20 wildtype (WT) and PN14 *Klf4*-conditional null (*Klf4*CN) mice, in which goblet cells are absent, developing, present, and missing, respectively. We identified 668, 251, 1160, and 139 transcripts that were increased and 492, 377, 1419, and 57 transcripts that were decreased between PN9 and PN14, PN14 and PN20, PN9 and PN20, and PN14 WT and *Klf4*CN conjunctiva, respectively [16]. Comparison of the conjunctival Klf4-target genes [16] with the corneal Klf4-target genes [66] identified a small number of common target genes, suggesting that Klf4 performs distinct functions in different tissues, by regulating a diverse array of targets. This tissue-selective nature of Klf4 is important in view of the widespread expression of Klf4 in several parts of the body. How such tissue-selective nature is achieved remains to be understood. 

Klf4 may exert its influence on conjunctival goblet cells directly or by controlling the expression of other transcription factors regulating goblet cell development. By comparing the wild type and *Klf4*CN PN14 conjunctival forniceal gene expression, we identified the transcription factors affected in the *Klf4*CN conjunctiva. Four among these factors were previously shown to be required for goblet cell development in other tissues such as colon or lung. Transcripts encoding Spdef, Foxa1, and Foxa3 that regulate goblet cell development and epithelium-specific Ets (ESE) transcription factor family members were increased during conjunctival development [16]. 

Sterile alpha motif- (SAM-) pointed domain containing Ets family protein (SPDEF) is a member of the ETS family of transcription factors. SPDEF contains an ETS DNA-binding domain at the C-terminal and a regulatory region consisting of the SAM-pointed domain at the N-terminal [67]. It preferentially interacts with ETS-binding sites with a core sequence GGAT. Spdef is required for goblet cell development in the intestine [63, 67] and tracheal/laryngeal submucosal glands as well as the conducting airway epithelium after allergen exposure [61, 62]. In transient transfection assays, both Klf4 and Klf5 stimulated mouse Spdef promoter activity (Gupta and Swamynathan, unpublished). However, Klf5 had a relatively greater effect, and cotransfection with Klf4 and Klf5 did not have any additional stimulatory effect, suggesting that Klf4 and Klf5 act through the same cis-elements in Spdef promoter (Gupta and Swamynathan, unpublished) (Figure 3).

Though the studies summarized above have given us a general picture of the goblet cell gene expression patterns, we are yet to understand what goes wrong in pathophysiological conditions which affect goblet cell densities. For example, changes in gene expression associated with goblet cell hyperplasia in allergic conjunctivitis and asthma or, alternatively, their absence in ocular cicatrizing pemphigoids remains to be identified. Additional studies in these directions are necessary to gain a better understanding of the genetic network of transcription factors which regulate goblet cell development and function in healthy and disease conditions.

## 7. MicroRNAs in the Ocular Surface

Endogenous noncoding microRNAs (miRNAs) regulate development and differentiation by binding to complementary sequences within the 3′ untranslated region (UTR) of target mRNAs, affecting the stability of target mRNAs and modulating their translation [68]. About 30% of the protein-coding genes in the vertebrate genome are estimated to be regulated by miRNAs. One miRNA can target hundreds of target mRNAs, and a given mRNA can be targeted by multiple miRNAs, resulting in increased complexity of gene regulation by miRNAs. In the eye, several miRNAs expressed in a distinct tissue and cell-type specific manner have been detected [69–71]. Most of the studies on miRNAs in the ocular surface are focused on the cornea. MiRNAs expressed in and important for the rest of the ocular surface remain to be examined.

In a comprehensive survey of miRNA expression in ocular tissues using microarray and RNA in situ hybridization, different ocular tissues exhibited notably distinct miRNA enrichment patterns [70]. Cluster analysis identified groups of miRNAs that showed predominant expression in specific ocular tissues. Targeted disruption of Dicer, a ribonuclease essential for miRNA processing, disrupted corneal epithelium stratification and whole eye development, providing evidence for the importance of miRNAs in eye development [72]. 

MiR184, one of the most well-studied miRNAs in the cornea, is abundantly expressed in the corneal epithelium. A mutation in miR-184 is responsible for familial keratoconus, a severe and painful corneal disorder [73]. Expression of miR-184 is detected in early eye development and corneal epithelial differentiation of human-induced pluripotent stem cells (hiPSCs) [74]. The knockdown of miR-184 resulted in a decrease in Pax6 and keratin-3, consistent with the observation that a point mutation in miR-184 results in corneal dystrophy [74]. In the first example of an miRNA negatively regulating another to maintain the levels of a target protein, the corneal epithelial-specific miR-184 was found to antagonize miR-205 that is widely expressed in the anterior segment epithelia and epidermis [69, 75]. miR-184 interferes with the ability of miR-205 to suppress the levels of lipid phosphatase SHIP2, thus maintaining its proper levels in the corneal epithelium [75].

Many other miRNAs play important roles in corneal development. Among them is miR-145, which regulates corneal epithelium formation and maintenance of epithelial integrity, by targeting the expression of integrin ITGB8 [76]. Another miRNA, miR-450b-5p, was identified as a molecular switch for Pax6 [74]. MiR-450b-5p and Pax6 are reciprocally distributed at the presumptive epidermis and ocular surface, respectively. MiR-450b-5p inhibited Pax6 expression and corneal epithelial fate in vitro, suggesting that miR-450b-5p triggers epidermal specification of the ectoderm by repressing Pax6. Thus, the absence of miR-450b-5p allows ocular epithelial development [74]. 

## 8. Transcription Factors Regulating Ocular Surface Development

**Figure 4:**
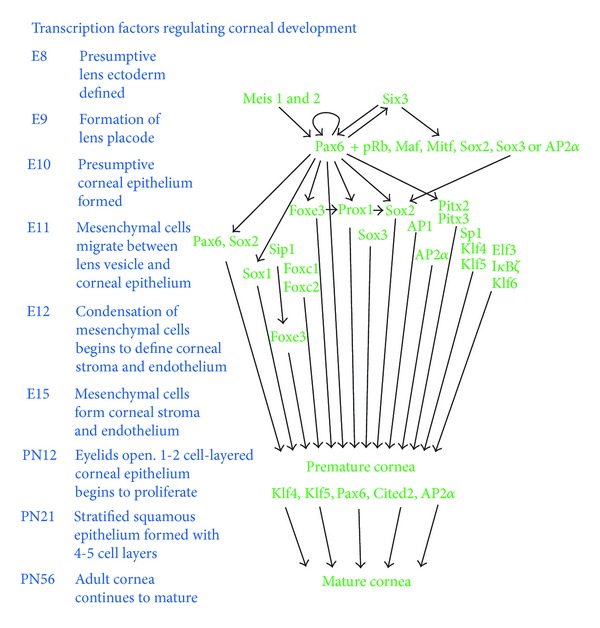
Transcription factors affecting corneal development. Pax6 serves as a master regulator of eye development in association with a handful of other transcription factors. While the involvement of several transcription factors has been studied in early embryonic development of the eye, few studies have focused on postnatal maturation of the cornea. Embryonic (E) or postnatal (PN) developmental stages (in days) are shown on the left.

Developmental studies utilizing transgenic and knockout technologies have revealed the contributions of a number of different transcription factors to mouse eye development (Figure 4). Human ocular surface developmental disorders associated with defects in genes encoding these transcription factors are consistent with their conserved roles in eye development across species. A brief review of the contribution of these transcription factors to maturation and maintenance of the ocular surface is provided below.

### 8.1. Homeobox Transcription Factors

#### 8.1.1. Paired Domain-Homeodomain Transcription Factor Pax6

Pax6, a paired domain-homeobox transcription factor, is considered the master regulator of eye development in view of its pivotal, highly conserved role in morphogenesis of the eye [77]. In the mouse, Pax6 expression is first detected at the optic pit, head surface ectoderm, and neural ectoderm on E8. After E13.5, *Pax6* is expressed in the proliferating anterior epithelial cells of the lens vesicle and the surface ectoderm which give rise to cornea, conjunctiva, and eyelids. The early embryonic expression of Pax6 in the developing eye is autoregulated by Pax6 and, in addition, by other homeobox transcription factors such as Meis1 and Meis2 (Figure 4) [78]. Pax6 activity is required in the lens primordium for lens formation and for correct placement of the retina in the eye [79]. In addition, *Pax6* is expressed throughout the ocular surface [80], where it plays a major role in early embryonic development and postnatal maturation of the corneal and conjunctival epithelia [81–83]. Though Pax6 is required for lacrimal gland development [13], its involvement in meibomian gland development is yet to be explored. In the adult mouse, *Pax6* continues to be expressed in the lens epithelial cells, cornea, conjunctiva, iris, ciliary body, and retina [80] and plays a major role in maintenance of the ocular surface. 

Pax6 is required for early embryonic development of the corneal epithelium, stroma, and endothelium [82, 83] as well as corneal innervation [84]. Mutations in *Pax6* result in severe defects in the human eye anterior segment [85–88]. Homozygous *Pax6* mutant mice develop only rudiments of the optic vesicle and die in the neonatal stage [89]. Corneal abnormalities in the heterozygous *Pax6* (*Small eye, Sey*) mouse mimic human aniridia-related keratopathy [90]. The lens placode formation is delayed in Pax6 heterozygous mice, resulting in a smaller lens frequently fused to the cornea, resembling Peter's anomaly [91]. The corneal epithelium in *Pax6*+/− (*Sey*) mouse is thinner with reduced number of cell layers despite increased cell proliferation, suggestive of increased epithelial erosion. The *Sey* mouse corneal epithelium contains decreased levels of desmoglein, *β*-catenin, *γ*-catenin, and keratin-12, consistent with defective intercellular adhesion [83]. In addition, the *Sey* mouse corneal epithelial cells have defective cell surface glycoconjugates that restrict their ability to migrate during wound healing [92]. Distribution of neural crest-derived cells is abnormal in *Sey* mouse, indicating that Pax6 regulates the normal distribution and integration of neural crest-derived cells in the mouse cornea [93].

Corneal activities of Pax6 are dosage dependent, as shown by the defective morphogenesis when Pax6 is either over- or underexpressed [94]. Overexpression of Pax6 in the mouse cornea affected corneal epithelial cell proliferation and homeostasis, resulting in signs of inflammation and neovascularization [95, 96]. An interaction between Pax6 dosage and hedgehog signaling is necessary for maintenance and regeneration of the corneal epithelium [97]. Aberrant Pax6 dosage results in an abnormal corneal stroma and endothelium as well, suggesting that proper Pax6 dosage is essential for normal morphogenesis of all layers of the mouse cornea [98].

Pax6 influences ocular surface development both directly by controlling the expression of different genes that play critical roles in the ocular surface and indirectly by controlling many other transcription factors including Six3, c-Maf, MafA/L-Maf, Prox1, Sox2, and Foxe3 that play important roles in the ocular surface (Figure 4) [3, 79, 99–105]. Compound heterozygous mice with mutations in both *Pax6* and *Gli3* develop more severe phenotype than *Gli3*+/− or *Pax6*+/− mutants alone [106], providing evidence for functional interaction of Pax6 with other transcription factors. Pax6 regulates the expression of *δ*Np63, a transcription factor which in turn regulates limbal epithelial stem cell (LESC) proliferation [54]. Pax6 regulates the expression of *Six3*, a homeobox transcription factor that influences eye development directly by regulating the expression of structural and metabolic genes required for eye formation and indirectly by reciprocally activating the expression of Pax6 [107–110].

*(1) Contributions of Pax6 towards Maintenance of the Mature Cornea.* Maintenance of the mature corneal epithelium involves continuous replication of a slow dividing population of stem cells that resides in specialized niche areas near palisades of Vogt in the corneoscleral junction called limbus [35]. The transiently amplifying cells derived from the LESCs differentiate as they migrate centripetally and apically and are eventually sloughed off at the surface [5, 35, 111]. Pax6 plays an important role in mature corneal homeostasis by helping maintain the limbal epithelial stem cell population. The limbal stem cells deficiency (LSCD) results in pterygium (characterized by in-growth of conjunctival cells and corneal neovascularization eventually resulting in corneal opacity) [112]. Similarly, LSCD is thought to cause aniridia, linked with human PAX6 gene mutations. The pathophysiology associated with aniridia-related keratopathy (ARK) is likely caused by LSCD, with associated defects in wound healing responses [112, 113]. 

Pax6 plays a critical role in adult corneal epithelial wound healing as well [94, 112]. *Pax6*+/− mice display many defects in corneal cell surface glycoconjugates and wound healing [92]. Pax6 influences corneal epithelial wound healing in association with hedgehog signaling [97]. Pax6 is elevated at the migrating wound epithelial edge where it upregulates gelatinase B (gelB; MMP-9) [114]. Pax6 influences target gene expression both independently and in association with other transcription factors such as pRb, MafA, MitF, Sox2, AP2*α*, and Sox3. Sox2 and Sox3 interact with Pax6, leading to synergistic transcriptional activation [99]. There are two Pax6 binding sites within the *gelB* −522/+19 bp promoter fragment [115]. Pax6 controls the *gelB* promoter activity by interacting directly with one of these sites and indirectly with the other site, through cooperative interactions with AP2*α* [114]. Overall, these studies indicate that Pax6 plays a significant role in embryonic development, postnatal maturation and maintenance of cornea.

#### 8.1.2. POU Homeodomain Transcription Factor Pitx2

Bicoid-related POU homeodomain transcription factor Pitx2, expressed in the neural crest, and the mesoderm-derived precursors of the periocular mesenchyme also contribute to the ocular surface development. In humans, *PITX2* mutations account for a large portion of the Axenfeld-Rieger malformations of the anterior segment [85]. In the mouse, deletion of *Pitx2* resulted in severe disruption of periocular mesenchyme structures and extrinsic defects in early optic nerve development. Pitx2 is required in neural crest for specification of the corneal endothelium and stroma and the sclera [116]. Corneal functions of Pitx2 also appear to be dosage dependent. *Pitx2* heterozygous mutant mice display reduced central corneal thickness [117], while overexpression of Pitx2a isoform in the mouse corneal mesenchyme and iris results in corneal opacification, corneal hypertrophy, and iridocorneal adhesions [118]. 

Pitx2 influences eye development through its involvement in retinoic acid signaling and Wnt signaling pathways which play integral roles in the periocular mesenchyme. Pitx2 activates Dkk2, an antagonist of canonical Wnt signaling, suppressing canonical Wnt pathway during eye development [119]. Retinoic acid signaling indirectly represses Wnt signaling in perioptic mesenchyme via induction of Pitx2 [120]. Thus, Pitx2 regulates early eye development by serving as a link in the crosstalk between retinoic acid signaling and Wnt signaling. The anterior segment expression of *Pitx2* is regulated by orphan G-protein-coupled receptor 48- (Gpr48/LGR4-) mediated cAMP-CREB signaling pathway [121].

### 8.2. Nonhomeobox Transcription Factors Regulating the Development of Cornea

#### 8.2.1. High Mobility Group Protein Hmgn1

The nucleosome binding high mobility group (HMGN) proteins are a group of nonhistone nuclear proteins that influence gene expression by altering the chromatin structure. Many different members of the Hmgn family proteins are expressed in the developing eye [122]. The *Hmgn1*-null mice develop thin, poorly stratified corneal epithelium depleted of suprabasal wing cells and a disorganized basement membrane, suggesting that Hmgn1 plays essential roles during mouse corneal epithelial development [123]. Epithelial cell-specific markers glutathione S-transferase- (GST-) *α*4 and -*ο*1 are reduced in *Hmgn1*-null corneas, while the components of adherens junctions—E-cadherin and *α*-, *β*-, and *γ*-catenin—are upregulated [123].

#### 8.2.2. Winged Helix/Forkhead Transcription Factors

Forkhead box (FOX) proteins are a family of transcription factors that share homology with the forkhead transcription factor in Drosophila [124] and play important roles in embryonic development by regulating the expression of genes involved in cell growth, proliferation, and differentiation [125, 126]. A unified nomenclature grouped the FOX proteins into subclasses (FOXA-FOXS) based on sequence conservation [127]. Many forkhead family members play important roles in normal development of different components of the ocular surface [59, 60, 128–140]. Among them, Foxc1 and Foxc2 have attracted the most attention in view of their association with Axenfeld-Rieger syndrome (ARS).

Foxc1 is the first forkhead factor to be associated with ocular surface development [133]. The expression of *Foxc1* gene is first detected in the periocular mesenchyme at E11.5 and is downregulated as the corneal endothelium differentiates [133]. *Foxc1*-null mice die at birth with multiple abnormalities including anterior segment dysgenesis involving corneolenticular fusion with a thicker corneal epithelium, disorganized stroma, and missing endothelium [131, 133, 141]. *Foxc1* heterozygous mice are viable with milder anterior segment defects [131, 139]. In humans, autosomal dominant mutations in *FOXC1* gene (in addition to those described above for *PITX2*) have been documented to cause anterior segment dysgenesis resembling ARS which affects additional parts of the body including the teeth and abdominal region [132, 142–145]. 

Corneal avascularity is an essential feature that ensures unimpeded transmission of light towards the retina. Recent studies have begun to unravel the well-conserved mechanisms that ensure corneal angiogenic privilege [146, 147]. Foxc1 plays an integral role in this regulation by controlling the normal delicate balance between factors in the cornea that promote angiogenesis and those that inhibit it. Interestingly, though Foxc1 is required for preserving mouse corneal transparency by inhibiting vascular growth [138], studies in human patients with *FOXC1* mutations contradict this finding [148], suggesting species-specific functions for Foxc1. 

Foxc1 and Foxc2 have nearly identical DNA binding domains, expression patterns, and functions in the developing eye [130, 139]. Mutations in *FOXC2* also resulted in ocular anterior segment anomalies, suggesting overlapping functions for these related factors [128, 134]. *Foxc1* and *Foxc2* double heterozygous mice have malformations of the ciliary body not seen in either heterozygous mouse alone [139]. Overlapping influence of forkhead family transcription factors FOXC1 and FOXC2 and the POU domain factor PITX2 described above may explain the variability and heterogeneity associated with the anterior segment dysgenesis in ARS [149, 150]. 

Forkhead box transcription factors also play a significant role in the development of other compartments of the ocular surface. Foxc1 is expressed in both the epithelium of the lacrimal gland and the surrounding mesenchyme. Foxc1 influences lacrimal gland development, as demonstrated by the severely impaired lacrimal glands in homozygous null Foxc1 mouse mutants with reduced outgrowth and branching [135]. 

Other related forkhead box transcription factors also influence anterior segment morphogenesis. For example, Foxe3 is required for anterior segment morphogenesis and differentiation in a Pax6 gene dosage-dependent manner [129]. Mutations in *Foxe3* are associated with defective lens development, and iridocorneal and iridolenticular fusions reminiscent of Peter's anomaly [151]. *Foxe3* expression in the eye is regulated by Msx2, a transcription factor implicated in anterior segment development [140]. A recent study identified a novel forkhead factor *Foxf2*, located near the *Foxc1* locus, as another candidate factor regulating anterior segment morphogenesis [136]. Heterozygote *Foxf2* mutant mice display thinner iris, hyperplasia of the trabecular meshwork, small or absent Schlemm's canal, and a smaller iridocorneal angle, while homozygous *Foxf2* mutant pups lack ciliary body projections at E18.5, suggesting a dosage-dependent role for Foxf2 in anterior segment morphogenesis [136].

#### 8.2.3. Sp1/Krüppel-Like Transcription Factors

Sp1/Krüppel-like transcription factors belong to the large family of zinc finger family proteins [152]. Several members of this family are expressed in the ocular surface in an overlapping manner [31, 153]. Members of the Sp1/KLF family possess divergent regulatory domains but similar DNA-binding domains, which enable them to bind similar cis-elements with comparable affinity, allowing fine regulation of their target genes in response to different stimuli. Here, I summarize the roles of Sp1/KLFs in ocular surface development and gene expression.

*(1) Specificity Protein Sp1.* Sp1 expression first detected in the head surface ectoderm as early as E10.5 continues in the cornea in the later stages [154]. However, Sp1 levels in the cornea decline gradually following eyelid opening. Within the cornea, Sp1 appears to be relatively more abundant in the basal cell layers and keratocytes. Expression of Sp1 is upregulated in a severe cornea-thinning disease called keratoconus [155, 156]. Sp1 is believed to play a role in keratoconus disease progression by supporting the increased expression of degradative enzymes such as cathepsin-B and suppression of proteinase inhibitors such as *α*1-proteinase inhibitor (*α*1-PI) [156, 157]. Downregulation of *α*1-PI in the corneal epithelium mediated by Sp1 may be a key event in keratoconus progression, supporting the possibility that the corneal epithelium also is involved in keratoconus, along with the stroma [158].

Sp1 is involved in regulation of several genes with important functions in the cornea. For example, Sp1 regulates corneal expression of keratin-3 in association with AP-2 [159, 160] and keratin-4 promoter activity in a cyclin D1-regulated manner [161]. Sp1 also activates corneal epithelial *involucrin* gene expression [162]. A recent study demonstrated that Sp1 activates expression of *α*5-integrin in association with another transcription factor, AP-1 [163]. Expression of Sp1 is elevated during wound healing, where it may be required for elevated expression of gelatinase-B (MMP9) [164].

*(2) Krüppel-Like Factor 4 (Klf4).* Krüppel-like transcription factor Klf4 is one of the most highly expressed transcription factors in the mouse cornea [31]. Klf4 is one of the four transcription factors (along with Oct3/4, Sox2, and c-Myc) required for generation of induced pluripotent stem cells from mouse embryonic or adult fibroblasts [165]. As each of these factors is present in the corneal limbus, it is likely that they are involved in the maintenance of limbal stem cells, the source of epithelial cells in the mature cornea.

Klf4 expression is detected in the ocular surface around E10 and is sustained in the adult cornea, increasing after eyelid opening. Conditional disruption of *Klf4* in the surface ectoderm-derived structures of the eye resulted in fragile corneal epithelium, swollen, vacuolated basal epithelial and endothelial cells, edematous stroma, and loss of conjunctival goblet cells [65]. Interestingly, Klf4 is also required for colonic goblet cell development [166], suggesting that similar networks may regulate goblet cell development in diverse mucosal epithelia. Stromal edema in *Klf4*CN corneas is associated with defective collagen fibril organization and increased degradation of stromal proteoglycans [167]. *Klf4*CN corneal stromal edema and epithelial fragility coupled with hypercellularity in these stromas suggested a proinflammatory environment in *Klf4*CN corneas [65]. However, the identity of these infiltrating cells remains to be established.

Studies using germline or conditional knockout mice demonstrated that Klf4 is required for both skin and corneal epithelial barrier function through regulation of expression of different components of the desmosomes [168, 169]. Direct involvement of Klf4 has been demonstrated in regulation of corneal epithelial expression of keratin-12, aquaporin-3, aquaporin-5, and corneal crystallins TKT and Aldh3a1 [65, 66]. Microarray comparison of WT and *Klf4*CN corneal and conjunctival gene expression patterns helped identify the potential Klf4-target genes in these two adjacent tissues of the ocular surface [16, 66]. Significant differences in conjunctival and corneal Klf4-target genes suggested tissue-dependent regulatory targets for Klf4 [16, 66].

*(3) Krüppel-Like Factor 5 (Klf5).* Klf5 is structurally related to Klf4 with an identical C-terminal DNA-binding domain capable of binding similar cis-elements and a divergent N-terminal regulatory domain that allows them to exert divergent influence on their target promoters [152]. Klf5 expression largely overlaps with that of Klf4, raising interesting questions related to their choice of target genes in a tissue where they are coexpressed. The ocular surface expression of *Klf5*, detected as early as E12, increases during postnatal stages [64]. Following apparently normal embryonic eye morphogenesis, *Klf5*CN corneas displayed defective postnatal maturation resulting in relatively smaller eyes containing translucent corneas with fragile epithelium, abnormal epithelial basement membrane, and edematous and hypercellular stroma [64]. In addition, *Klf5*CN eyelids were malformed with defective meibomian glands. *Klf5*CN conjunctiva lacked goblet cells, suggesting that Klf5 is required for conjunctival goblet cell development. Klf5 is also required for lung goblet cell development, suggesting that similar transcriptional networks regulate goblet cell development in diverse mucosal epithelia [64, 170].

Severe phenotype observed when either *Klf4* or *Klf5* was disrupted in the ocular surface, keeping the other gene intact, suggested nonredundant functions for these two structurally related factors. Consistent with this, simultaneous co-ablation of both *Klf4* and *Klf5* resulted in a more severe ocular surface phenotype compared with *Klf4*CN or *Klf5*CN, demonstrating that Klf4 and Klf5 share few, if any, redundant functions [64]. Finally, comparison of corneal Klf4- and Klf5-target genes revealed that roughly one-third of the target genes are unique to each factor, with the other third being shared, common target genes [66].

*(4) Krüppel Like Factor 6 (Klf6).* Klf6 expression is detected in the head surface ectoderm as early as E10 and in the corneal epithelium and stroma around E15.5, and it increases in postnatal stages [171]. Klf6 also binds and activates keratin-12 (Krt12), an intermediate filament required for corneal epithelial homeostasis [172]. However, it is not clear if Klf4 and Klf6 activate Krt12 through the same cis-elements, or if they target different regions of the Krt12 promoter. Interestingly, KLF6 expression also is elevated in keratoconus, a progressive disease associated with thinning and scarring of the cornea [173]. A likely explanation for the involvement of KLF6 in keratoconus may be found in the fact that KLF6, like Sp1 described above, downregulates the *α*1-*proteinase inhibitor* (**α*1-PI*) gene in corneal epithelial cells [173].

#### 8.2.4. Activating Protein (AP) Family

*(1) Activating Protein-1 (AP1).* AP1 consists of a group of basic leucine zipper (bZip) family of dimeric complexes formed by the various Jun, Fos, Fra, and ATF proteins. Members of the AP1 family selectively bind the tetradecanoylphorbol acetate- (TPA-) responsive element (TRE; 5′-TGAG/CTCA-3′) and activate transcription of nearby promoters. AP1 family members regulate cell proliferation in response to various stimuli. Many AP1 family members are expressed in the cornea (Figure 4) [31, 174–177]. AP1 is necessary for expression of involucrin, a structural protein that is selectively expressed in differentiating corneal epithelial cells [162, 177]. In transgenic mice, removal of the AP1 site by truncation or point mutation results in a loss of *involucrin *expression, confirming the importance of AP1 for involucrin promoter activity during corneal epithelial cell differentiation.

*(2) Activating Protein-2 (AP-2).* The activating protein-2 (AP-2) family of transcription factors, consisting of five different members AP-2*α*, AP-2*β*, AP-2*γ*, AP-2*δ*, and AP-2*ε*, stimulate proliferation and suppress terminal differentiation in a cell-type-specific manner during embryonic development [178]. Corneal epithelial expression of AP-2*α* is confined to basal epithelial cells while AP-2*β* is broadly expressed throughout all cell layers [179–181]. AP-2*α* is also highly expressed in the less differentiated cell layers of the eyelid epidermis [179]. *Ap-2α* null embryos exhibit a range of phenotypes from a complete lack of eyes to defective lens attached to the overlying surface ectoderm [181]. Conditional deletion of *Ap-2α* in lens placode derivatives, including the corneal epithelium, results in a decrease in the expression of the cell-cell adhesion molecule E-cadherin, misexpression of laminin, entactin and type IV collagen, and disruption of stromal collagen fibril organization, showing that AP-2*α* is required for proper formation of the mouse cornea [179, 182]. Pax6 and AP-2*α* interact with each other and coordinate the expression of gelatinase-B (matrix metalloproteinase 9) and corneal epithelial repair [114].

#### 8.2.5. Ets Family Members

The Ets transcription factors belong to a large family comprising of 29 related genes in humans (28 in the mouse) named after E-twenty six (E26), a gene transduced by the leukemia virus. Ets family members are expressed throughout the body and are implicated in development and cancer progression. Ets family transcription factors are involved in regulation of a wide variety of functions including cell cycle control, cell differentiation, cell migration, cell proliferation, apoptosis, and angiogenesis. Ets family transcription factors are characterized by a conserved winged helix-turn-helix DNA-binding domain that binds the consensus DNA sequence 5′-GGA(A/T)-3′. Additional sequence specificity is achieved through interaction with other cofactors and the neighboring sequence. Ets family members often influence gene expression in cooperation with other transcription factors, which make their effects more versatile. For example, Ets-1 and Ets-2 cooperate with the AP-1 transcription factor, while Elk-1 and SAP-1 cooperate with the serum response factor (SRF).

The protooncogene *Ets*-1, which plays a key role in angiogenesis and matrix degradation, is upregulated in many cases of pterygial angiogenesis [183]. An Ets family epithelium-specific transcription factor Ese-1/Elf3 is upregulated in differentiating mouse corneal epithelium and in immortalized human corneal epithelial (HCE) cells, and transactivates *keratin-12* through Ets-binding sites [184]. Suppression of Ese-1/Elf3 expression by antisense RNA in HCE cells affects their differentiation, providing evidence for the involvement of Ese-1/Elf3 in differentiation of corneal epithelial cells (Figure 4).

#### 8.2.6. Transcription Factors Regulating Hypoxic Stress Response in the Cornea

When the eyelids are closed during sleep, the avascular cornea is subjected to almost 75% drop in oxygen partial pressure [185, 186]. Thus, hypoxic and xenobiotic response pathways are essential for proper maintenance of corneal homeostasis. The important role of inhibitory PAS (IPAS) domain protein—a hypoxia repressor protein—in maintaining corneal avascularity [187, 188] is consistent with this prediction. *IPAS* gene expression is stimulated by hypoxia inducible factor-1*α* (HIF-1*α*) demonstrating a negative feedback regulatory circuit [188]. Additional evidence for the involvement of hypoxic and xenobiotic stress in regulating corneal gene expression comes from the fact that corneal crystallin genes are induced by hypoxia or xenobiotics [189–191]. We demonstrated that the xenobiotic metabolism-related pathways are significantly enriched among genes whose expression is decreased in *Klf5*CN corneas, suggesting that Klf5 serves an important role in detoxification of the environmentally exposed avascular cornea [32]. Other transcription factors such as NF-*κ*B, Klf5, Cited2, and CTCF are implicated in regulating hypoxia-related gene expression and are discussed below.

*(1) NF-κ* *B and the Related Factors.* NF-*κ*B plays an essential role in corneal wound healing process. NF-*κ*B is up-regulated in the early stage of cauterization induced corneal neovascularization (CNV), suggesting that it participates in wound healing, inflammation, and neovascularization in the cornea [192]. NF-*κ*B is activated in corneal pathologies involving increased plasma levels of LPS and Tumor Necrosis Factor-*α* (TNF-*α*), as well as direct UV-B exposure [193]. TNF-*α* disrupted the barrier function of cultured human corneal epithelial cells in an NF-*κ*B-dependent manner [194]. Thymosin-*β*4 suppresses NF-*κ*B activity, providing evidence suggesting that its anti-inflammatory effects are mediated through NF-*κ*B [195, 196]. 

NF-*κ*B plays important roles in resolving corneal viral infections. Herpes simplex virus- (HSV-) 1 infection of the human cornea first induces and then blocks epithelial cell apoptosis in an NF-*κ*B-dependent manner [197]. NF-*κ*B is activated in respiratory syncitial virus-infected corneal epithelial cells [198]. NF-*κ*B activation is partly responsible for the acute inflammation in adenoviral-infected corneas [199]. NF-*κ*B and PI3K-Akt signaling pathways mediate the poly(I:C-) induced VCAM-1 and ICAM-1 upregulation in corneal fibroblasts, thus regulating the corneal stroma inflammatory responses to viral infection [200]. 

NF-*κ*B pathway is rather complex and is regulated by several inhibitors, which also play a role in regulating corneal homeostasis. I-*κ*K*α* is required for formation of cornea and conjunctiva, possibly due to its ability to regulate NF-*κ*B activity [201]. I-*κ*B-*ζ* is another regulator of the transcription factor NF-*κ*B and is expressed in the ocular surface epithelium, a part of the mucosal defense system [202]. The pathologic progression of ocular surface inflammation is inhibited by I-*κ*B-*ζ* [202], as demonstrated by the chronic inflammation in *I*-*κB*-*ζ*-null mouse ocular surface accompanied by loss of conjunctival goblet cells [202].

*(2) Cited2.* Cited2 is an acronym for “Cbp/p300-interacting transactivator, with Glu/Asp-rich carboxy-terminal domain-2.” Cited2 is a transcriptional coactivator in the p300/CBP-mediated transcription complex. It acts as a bridge linking p300/CBP transcriptional coactivator complex with TFAP2 transcription factors, stimulating TFAP2-target gene promoter activities. Cited2 acts as a positive regulator of TGF-*β* signaling through SMAD/p300/CBP-mediated transcriptional coactivator complex. CITED2 ectopic expression attenuated the expression of NF*κ*B-responsive genes, while CITED2 knockdown stimulated the NF*κ*B-responsive genes, suggesting that Cited2 acts as a negative regulator of NF*κ*B [203]. Mechanistic basis of this action was revealed by the discovery that CITED2 prevented p300-mediated acetylation of p65 subunit of NF*κ*B, thus attenuating p65 binding to its cognate promoters [203].

Conditional disruption of *Cited2* resulted in severe ocular surface defects including corneal opacity and spontaneous corneal neovascularization at older age [204]. In the absence of Cited2, there were fewer layers of corneal epithelial cells that lacked Krt12 expression. Cited2 deficient corneal epithelial debridement wound healing was impaired. Additional gene expression studies indicated that Cited2 may influence corneal morphogenesis by modulating the expression of *Pax6* and *Klf4* [204]. CITED2 negatively regulates the expression of hypoxia-responsive genes. CITED2 serves as a negative regulator of hypoxia-inducible factor-1 (HIF-1) by interacting with the p300/CBP CH1 domain, disrupting the formation of the HIF-1 and p300/CREB-binding protein (p300/CBP) heterodimeric complex [205].

*(3) CCCTC-Binding Factor (CTCF).* CTCF is an epigenetic transcription factor containing a 11-zinc finger DNA-binding domain [206]. CTCF is considered a key player in insulator function, which isolates the effects of cis-elements from spreading beyond the intended loci in eukaryotic genomes. The presence of 11 zinc fingers provides this factor with an ability to interact with a host of different target sites, increasing its functional versatility [207].

CTCF is believed to play a role in epidermal growth factor-(EGF-) mediated suppression of Pax6 accompanying elevated cell proliferation in corneal epithelial cells [208]. Overexpression of CTCF suppressed Pax6 P0 promoter activity through CTCF binding elements located around −1.2 kb upstream of the P0 promoter [209, 210]. Expression of CTCF is regulated by epidermal growth factor (EGF) through activation of NF-*κ*B. CTCF participates in stress-induced signaling cascades, playing a significant antiapoptotic role in cultured human corneal epithelial cells [211]. Oxidative stress activated Bcl3, which in turn suppressed the expression of CTCF through a noncanonical NF-*κ*B pathway [212, 213]. Recent evidence indicates that hypoxic stress induces de-SUMOylation of CTCF to functionally regulate its activity [214].

## 9. Actin Cytoskeleton Remodeling and Corneal Gene Expression

Severe corneal abnormalities associated with mutations in actin cytoskeleton remodeling-related proteins such as destrin and KLEIP suggested an essential role for actin in corneal homeostasis [215–222]. Scinderin, an actin depolymerizing protein similar to gelsolin, is abundantly expressed in Zebrafish and Anableps corneas [223–226]. Interestingly, mutations in gelsolin are associated with the Finnish type of familial amyloidosis, a systemic disease characterized by corneal lattice dystrophy, progressive cranial neuropathy, and distal sensorimotor neuropathy in humans [227, 228]. The amyloid subunit in Finnish hereditary amyloidosis is derived from the actin filament-binding region of gelsolin, suggesting the involvement of defective regulation of actin dynamics [229, 230].

The BTB-kelch domain protein KLEIP regulates cell-cell contact formation and cell migration [215]. *KLEIP*−/− mouse corneas develop normally, but display corneal epithelial hyperplasia and progressive metaplasia leading to total corneal opacity in post-eyelid opening stages [215]. *KLEIP*−/− corneal stroma was heavily neovascularized and infiltrated with numerous cells [215]. *KLEIP*−/− corneal epithelium was altered to an epidermal-like structure with superficial keratinized cells and expression of skin markers keratin-1 and loricrin [215]. Molecular mechanism(s) underlying the dramatic phenotype in *KLEIP*−/− corneas remain(s) to be identified.

Another actin-binding protein implicated in regulating corneal homeostasis is destrin (Dstn), a member of the ADF/cofilin family of proteins that regulates actin dynamics by depolymerizing filamentous actin into monomeric form. A spontaneous mouse mutant in *Dstn* (corneal disease 1 or Corn1) causes epithelial hyperproliferation and neovascularization [219]. Corn1 mouse cornea is characterized by irregular thickening of the epithelium with increased filamentous actin content, presence of actin stress fibers, and signs of autoinflammatory condition such as increased influx of neutrophils, elevated expression of Cxcl5, and neovascularization [217, 219, 221]. *Dstn* mutations and resultant changes in actin dynamics have a strong influence on corneal gene expression profile [216]. Microarray analysis revealed dramatic alteration in Corn1 mouse corneal gene expression profile [216]. Genes associated with actin cytoskeleton dynamics were among the most significantly enriched along with a number of serum response factor target genes, suggesting that actin cytoskeleton dynamics regulates SRF-mediated transcriptional control [216, 222]. Consistent with this, conditional ablation of Srf in the *Dstn*−/− corneal epithelium rescued Corn1 corneal phenotypes including epithelial cell hyperproliferation, inflammation, and neovascularization, confirming an epithelial cell-specific role for SRF [222].

## 10. Regulation of Expression of Genes with Important Functions in the Cornea

### 10.1. Corneal Crystallins

A common assumption in many studies on gene regulation is that gene expression is controlled mainly at the level of transcription. However, post-transcriptional regulation appears to play a significant role in the expression of corneal crystallins aldehyde dehydrogenase IIIA1 (Aldh3a1) and transketolase (Tkt), which constitute roughly 50% and 10% of the water-soluble protein, respectively, and only about 1% each of the total mRNA in the adult cornea [31]. It remains to be determined if this regulation is achieved through increased stability of these specific transcripts and/or their selective overtranslation.

The corneal crystallin *Aldh3a1* is expressed at about 500-fold higher level in the mouse corneal epithelial cells than in other tissues [231]. Corneal expression of *Aldh3a1* is temporally regulated, increasing by about 100-fold between birth and 6 weeks of age. A 4.4 kb mouse Aldh3a1 promoter fragment was shown to regulate *Aldh3a1* expression in the corneal epithelial cells in transgenic mice, suggesting that *cis*-elements for corneal expression reside within this fragment [231]. The high level of ALDH3 expression involves a strong basal promoter region and a xenobiotic response element (XRE) located within −3.0 kb [191]. ARNT, HNF1, and HNF4 interact with the ALDH3-XRE in an aryl hydrocarbon-receptor-independent, ARNT-requiring manner to influence Aldh3a1 expression [191]. Aldh3a1 promoter activity is controlled synergistically by Pax6, Oct1, p300, KLF4, and KLF5 [66, 232]. A suppressor sequence resides within the first intron of the mouse *Aldh3a1* gene, although it is not known how it might influence endogenous gene expression [232]. Unlike the mouse, the rabbit corneas preferentially express aldehyde dehydrogenase class 1 (ALDH1A1) [11, 233]. The −3519 to +43 bp rabbit Aldh1a1 promoter fragment which contains three xenobiotic response elements (XREs) and one E-box element recapitulated this preferential expression in transgenic mice [190]. The hypoxia-inducible factor 3*α*/aryl hydrocarbon nuclear translocator heterodimer is implicated in *Aldh1a1* promoter activation via the XREs [190]. 

The second major crystallin in the mouse corneas is transketolase (Tkt) [234]. *Tkt* mRNA levels increase six-fold in the mouse cornea within 1-2 days of eyelid opening, in a manner dependent on exposure to light and oxidative stress [235]. Two transcription initiation sites separated by 630 bp that shares a common initiator ATG codon have been identified in *Tkt* gene [234, 235]. The proximal GC-rich transcription initiation site (within intron 1) lacking a TATA box is used for high corneal expression, while the distal transcription initiation site is used weakly in liver. Not much is known about the transcription factors regulating *Tkt* promoter activity, other than the involvement of Klf4 [66]. 

### 10.2. Keratins

Keratin-12 (Krt12), one of the more than 30 different keratins (intermediate filament components), is abundantly expressed specifically in the stratified corneal epithelium [12]. Heterozygous mutations in KRT12 cause Meesmann's corneal dystrophy, an autosomal dominant disorder that affects corneal epithelium. Disruption of *Krt12* gene results in fragile corneal epithelium resembling Meesmann's corneal dystrophy in the mouse [236]. From E15.5 to PN10, Krt12 expression is restricted to the suprabasal and/or superficial cells of the corneal epithelium. After PN30, the number of Krt12-positive basal cells increases with age [12]. Particle-mediated gene transfer helped identify corneal epithelial-specific cis-elements within a 2.5 kb *Krt12* promoter fragment [237]. Pax6, Klf4 and Klf6 stimulate Krt12 promoter activity [65, 66, 172, 238]. Expression of *Krt12* is delayed and downregulated in the *Pax6*+/− corneal epithelium, implying regulation of Krt12 promoter activity by Pax6 [113]. Interestingly, overexpression of Pax6 also affected Krt12 expression, providing evidence for a tight dosage-dependent influence of Pax6 on *Krt12* promoter activity [95].

### 10.3. Lumican

Lumican (Lum), a member of the small leucine-rich proteoglycan (SLRP) family that includes decorin, biglycan, fibromodulin, and keratocan [239], is the major keratan sulfate proteoglycan (KSPG) of the cornea. In addition to its high level of expression in the cornea, lumican is also expressed in most interstitial collagenous matrices, including the connective tissues of the heart [240]. Though lumican mRNA is detected early during embryogenesis in the cornea and sclera, the mature sulfated KSPG core proteins can be detected only after the eyes open by PN12 [240]. 

Lumican is an important determinant of corneal transparence as shown by the corneal opacity in lumican null mice [241]. Lumican regulates collagen fibril organization, corneal circumferential growth, neutrophil migration in response to bacterial infections, and epithelial cell migration during wound healing and tissue repair [30, 131, 239, 241–247]. Mechanistically, lumican protein binds collagen fibrils allowing the highly charged hydrophilic glycosaminoglycans (GAGs) to regulate interfibrillar spacing, an important determinant of corneal transparence [239, 242, 245]. In spite of its importance, not much is known about the regulation of lumican expression in the cornea. 

### 10.4. Keratocan

Keratocan (Kera) is another important KSPG abundantly expressed in the mouse corneal stroma. In humans, mutations in KERA gene are associated with cornea plana (CNA2) characterized by flattened cornea leading to decreased visual acuity. *Kera*−/− mice exhibit less severe phenotype than the *Lum*−/− mice [248, 249]. Though *Kera*−/− corneal stroma was thinner with disorganized and relatively thicker collagen fibrils, its corneal transparence appeared normal, suggesting that the contributions of keratocan towards maintenance of stromal collagenous matrix and corneal transparence are subtle, if any [249].

The expression of *Kera* is first detected in the periocular mesenchymal cells at E13.5. After E14.5, *Kera* expression is restricted to the stromal keratocytes [250]. In adult transgenic mice, expression of *β*-*geo* transgene driven by *Kera* 3.2 kb upstream sequence, exon 1, and 0.4 kb of intron 1, *β*-Gal activity was detected only in cornea. Spatiotemporal activity of this transgene recapitulated that of endogenous *Kera*, suggesting that the 3.2 kb upstream sequence contains the necessary *cis*-elements to regulate *keratocan* gene expression [251]. Interestingly, lumican and keratocan expressions appear to be coupled, as demonstrated by the increased expression of Kera upon overexpression of lumican and the reduced expression of Kera in Lum−/− mice [248].

## 11. Remaining Challenges and Future Prospects

We have witnessed substantial progress in our understanding of gene expression in the ocular surface. We have a good understanding of the expression and function of different transcription factors in regulating gene expression in the ocular surface. However, much of this understanding is restricted to the cornea, with a limited awareness of gene expression in the conjunctiva, lacrimal glands, and meibomian glands. In order to develop novel methods of diagnosis and pharmacotherapeutic intervention in ocular surface disorders, it is necessary to gain complete understanding of the changes in gene expression in the ocular surface and the mechanisms by which they are regulated. In view of the importance of the tear film in ocular surface health, it will be worthwhile studying regulation of gene expression in the lacrimal glands and meibomian glands which contribute the aqueous and lipid layers of the tear film, respectively, in greater detail. Similarly, molecular mechanisms governing the regulation of expression of mucins in the conjunctival goblet cells and corneal epithelial cells are understudied and deserve further exploration [58]. 

Four additional areas of research in ocular surface biology stand out as deserving extra attention in the immediate future. First, we have only recently begun to appreciate the depth of the contributions of miRNAs to ocular surface gene regulation and can expect many rapid and exciting new discoveries in this field in the coming years. Second, reliable markers that enable reproducible isolation of corneal limbal epithelial stem cells in a clinical setting have been elusive in spite of the tremendous effort from different laboratories over the last decade, and our effort in this direction needs to be stepped up. Third, changes in expression of the ocular surface and tear film specific markers in response to various proinflammatory chemical, physical, and microbial insults and the specific network of transcription factors responsible for these changes need to be characterized in great detail, facilitating improved diagnosis and therapeutic intervention in ocular surface disorders. Finally, most of the early large-scale ocular surface gene expression studies have focused on the transcript levels. However, multiple studies suggest a significant discordance between transcript and protein levels in a given cell, indicating that regulation at the level of alternate splicing, transcript stability, efficiency of translation, protein stability, and so forth, also play critical roles in gene expression (reviewed in [252, 253]). With the advent of large scale proteomics technologies in the near future, we can expect to uncover if the current findings at the transcript levels truly reflect corresponding protein levels.

Many studies described above have utilized advanced transgenic mouse technologies, germline deletions, and/or conditional inactivation of specific genes in the surface ectoderm-derived tissues of the eye. Further improvements in our ability to delete or mutate specific genes in a precise spatiotemporally regulated manner will further aid this effort. We can look forward with excitement to the identification of additional roles for the transcription factors currently known to influence ocular surface development. In addition, we can expect to discover the contributions of novel transcription factors and their interactions with other factors, revealing the molecular basis of regulation of gene expression in the ocular surface. Finally, ongoing improvements in instrumentation and techniques which facilitate collection of specific subsets of cells from hard-to-reach areas of the eye, coupled with the progress in second generation sequencing-based technologies such as RNA-Seq and ChIP-Seq which facilitate large scale transcriptome analyses at a relatively low cost, are expected to revolutionize our understanding of gene expression in the ocular surface.
